# Real-time PCR for malaria diagnosis and identification of *Plasmodium* species in febrile patients in Cubal, Angola

**DOI:** 10.1186/s13071-024-06467-3

**Published:** 2024-09-11

**Authors:** Alejandro Mediavilla, Aroa Silgado, Begoña Febrer-Sendra, Beatriz Crego-Vicente, Patricia Martínez-Vallejo, Carles Rubio Maturana, Lidia Goterris, Arlette Nindia, Joan Martínez-Campreciós, Sandra Aixut, María Luisa Aznar-Ruiz-de-Alegría, Pedro Fernández-Soto, Antonio Muro, Fernando Salvador, Israel Molina, Pedro Berzosa, Inés Oliveira-Souto, Elena Sulleiro

**Affiliations:** 1 Microbiology Department, Vall d’Hebron University Hospital, Autonomous University of Barcelona, PROSICS Barcelona, Barcelona, Spain; 2 Universitat Autònoma de Barcelona (UAB), Barcelona, Spain; 3 Centro de Investigación Biomédica en Red de Enfermedades Infecciosas (CIBERINFEC), Instituto de Salud Carlos III, Madrid, Spain; 4 Infectious and Tropical Diseases Research Group (e-INTRO), Biomedical Research Institute of Salamanca-Center for Research in Tropical Diseases of the University of Salamanca (IBSAL-CIETUS), Faculty of Pharmacy, University of Salamanca, Salamanca, Spain; 5 Hospital Nossa Senhora da Paz, Cubal, Angola; 6 International Health Unit Vall d’Hebron-Drassanes, Infectious Diseases Department, Vall d’Hebron University Hospital, PROSICS Barcelona, Barcelona, Spain; 7 Malaria and Neglected Tropical Diseases Laboratory, National Centre for Tropical Medicine, Carlos III Health Institute, CIBER de Enfermedades Infecciosas, Madrid, Spain

**Keywords:** Malaria, Angola, *Plasmodium*, Diagnosis, Real-time PCR, Species identification

## Abstract

**Background:**

Malaria is the parasitic disease with the highest morbimortality worldwide. The World Health Organization (WHO) estimates that there were approximately 249 million cases in 2022, of which 3.4% were in Angola. Diagnosis is based on parasite identification by microscopy examination, antigen detection, and/or molecular tests, such as polymerase chain reaction (PCR). This study aimed to evaluate the usefulness of real-time PCR as a diagnostic method for malaria in an endemic area (Cubal, Angola).

**Methods:**

A cross-sectional study was carried out at the Hospital Nossa Senhora da Paz in Cubal, Angola, including 200 patients who consulted for febrile syndrome between May and July 2022. From each patient, a capillary blood sample was obtained by finger prick for malaria field diagnosis [microscopy and rapid diagnostic test (RDT)] and venous blood sample for real-time PCR performed at the Hospital Universitario Vall d’Hebron in Barcelona, Spain. Any participant with a positive result from at least one of these three methods was diagnosed with malaria.

**Results:**

Of the 200 participants included, 54% were female and the median age was 7 years. Malaria was diagnosed by at least one of the three techniques (microscopy, RDT, and/or real-time PCR) in 58% of the participants, with RDT having the highest percentage of positivity (49%), followed by real-time PCR (39.5%) and microscopy (33.5%). Of the 61 discordant samples, 4 were only positive by microscopy, 13 by real-time PCR, and 26 by RDT. *Plasmodium falciparum* was the most frequent species detected (90.63%), followed by *P. malariae* (17.19%) and *P. ovale* (9.38%). Coinfections were detected in ten participants (15.63%): six (60%) were caused by *P. falciparum* and *P. malariae*, three (30%) by *P. falciparum* and *P. ovale*, and one (10%) triple infection with these three species. In addition, it was observed that *P. falciparum* and *P. malariae* coinfection significantly increased the parasite density of the latter.

**Conclusions:**

RDT was the technique with the highest positivity rate, followed by real-time PCR and microscopy. The results of the real-time PCR may have been underestimated due to suboptimal storage conditions during the transportation of the DNA eluates. However, real-time PCR techniques have an important role in the surveillance of circulating *Plasmodium* species, given the epidemiological importance of the increase in non-*falciparum* species in the country, and can provide an estimate of the intensity of infection.

**Graphical abstract:**

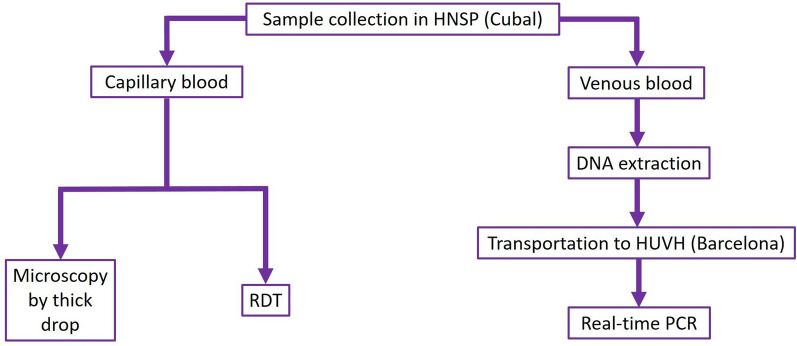

## Background

Malaria is the parasitic disease with the highest morbidity and mortality worldwide. According to the World Health Organization (WHO), approximately 249 million cases of malaria and 608,000 deaths were recorded worldwide in 2022 [1]. This anopheline mosquito-borne disease is caused by protozoa of the genus *Plasmodium*. The five species that infect humans are *Plasmodium falciparum*, *P. vivax*, *P. malariae*, *P. ovale* (subspecies: *P. ovale wallikeri* and *P. ovale curtisi*), and *P. knowlesi* [2].

Angola, located in southwest Africa, is one of the malaria high-burden countries, recording 3.4% of malaria cases and 3.2% of global malaria deaths in 2022 [1]. *Plasmodium falciparum* is the most prevalent species, however, molecular studies carried out in the last two decades have demonstrated the presence of *P. malariae*, *P. ovale,* and *P. vivax* in smaller proportions [3]. The malaria prevalence in this country varies according to latitude: the north is hyperendemic, the center is stable mesoendemic, and the south is unstable mesoendemic [4]. Cubal is a rural locality in the province of Benguela, located in central-western Angola, which has a tropical climate with a rainy season (November–May) associated with a period of high transmission and a dry season (July–October) of low transmission [5]. Malaria transmission in Cubal has been defined as stable mesoendemic in previous studies [6].

Accurate and early diagnosis of malaria is of great importance for effective treatment and correct management of the disease. The main diagnostic methods for malaria are based on the identification of parasitic forms by microscopic examination of blood smears, detection of antigens by rapid diagnostic tests (RDTs), or nucleic acids by molecular methods, such as polymerase chain reaction (PCR) and loop-mediated isothermal amplification (LAMP) [7]. Clinical diagnosis is widely used in endemic areas [8], however, symptoms are often not specific (fever, chills, muscle pains, headaches, and so on), and vary according to the stage of the disease or the different *Plasmodium* species causing the infection [9]. Therefore, the Global Technical Strategy for Malaria 2016–2030, adopted by the WHO Global Malaria Program in 2015, recommends that clinical suspicions of malaria should be confirmed by parasitological laboratory testing (microscopy and/or RDT) before treatment [10].

Microscopic examination remains the gold standard technique for malaria diagnosis as it allows for the quantification and identification of *Plasmodium* species in blood samples. However, microscopy has a highly expert-dependent detection limit of approximately 50–100 parasites/µL, which implies a low sensitivity in patients with a low parasite load and in those with coinfections of two or more *Plasmodium* species cases [8, 11, 12]. Further, RDTs are a simple, rapid, easy-to-perform, and cost-effective method for malaria diagnosis. The most frequent RDT targeting histidine-rich protein 2 of *P. falciparum* (*Pf*HRP2) has a high sensitivity when the parasite density is > 100 parasites/µL (94.3%), however, when parasitaemia is less than < 100 parasites/µL its sensitivity decreases considerably (74.1%) [13]. Moreover, RDTs do not allow quantification of the parasitaemia or accurate identification of the *Plasmodium* species, and mutations in the gene encoding the *Pf*HRP2 can lead to false-negative results [14, 15].

Currently, molecular methods, mainly based on PCR protocols, serve as relevant tools for the diagnosis of malaria due to their high sensitivity and specificity, as they are able to detect the presence of parasite DNA in asymptomatic patients or those with very low parasite loads (detection limit < 1 parasite/µL) [16, 17]. Moreover, PCR allows for the specific identification of *Plasmodium* species [18]. Quantitative real-time PCR enables the quantification of the parasite load [19], making it possible to monitor the response to treatment over time and establish the possible effect of coinfections on parasite density [20, 21]. However, PCR has not been efficiently implemented in resource-limited settings, mainly due to the high cost of the equipment and the need for laboratories and specialized technicians [22]. On the contrary, LAMP is a rapid and simple molecular method for the diagnosis of malaria in all types of endemic and nonendemic areas. In addition, the reagents and instrumentation used are cheaper than those required for PCR techniques [23].

The objective of this study was to evaluate the usefulness of real-time PCR for diagnosing malaria in patients with febrile symptoms in Cubal, Angola, comparing it with other diagnostic methods (microscopy and RDT).

## Methods

### Study population and data collection

A cross-sectional study was conducted at the Hospital Nossa Senhora da Paz (HNSP, Cubal, Angola) in febrile patients between May and July 2022. Recruitment of participants and sample collection was performed as described by Febrer-Sendra et al. [23], with febrile syndrome as the sole inclusion criterion.

Demographic data (age and gender) were collected for each participant. In terms of age, four population groups were established: preschool-aged children (PSAC) (0–4.9 years), school-aged children (SAC) (5–14.9 years), adults (15–49.9 years), and the elderly (> 50 years).

### Sample size calculation

The sample size was calculated from data published in a previous study at the HNSP of Cubal by Salvador et al. [6]. A sample size of 186 patients with suspected malaria was initially estimated with 95% confidence and a 5% accepted error, with the final sample size rounded to 200 participants [24].

### Sample collection and malaria diagnosis

**Fig. 1:**
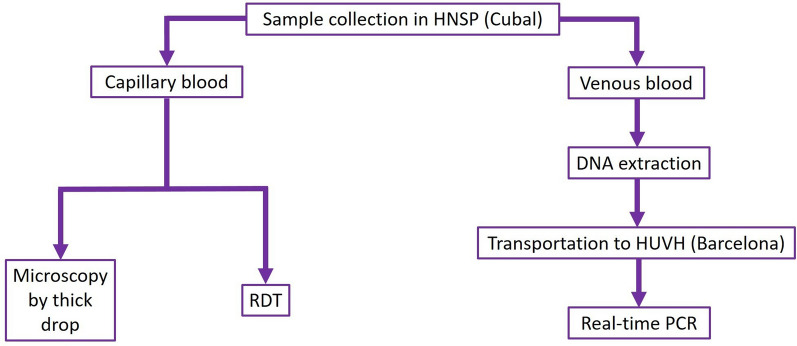
Flow chart established for sample collection and diagnostic methods used. Sample collection was carried out at the Hospital Nossa Senhora da Paz (HNSP) in Cubal. For real-time PCR, a previous DNA extraction step was required, the eluates of which were transported to the Hospital Universitario Vall d'Hebron (HUVH) in Barcelona

A capillary blood sample and a 3 mL venous blood sample (collected in a tube with EDTA anticoagulant) were collected from each participant following the clinical protocol established by the HNSP. Capillary blood obtained by finger stick was used for microscopic examination and RDT, whereas venous blood obtained by venipuncture was used for molecular analysis (Fig. 1).

Microscopy was used as the standard for diagnosing malaria. Until proven otherwise, it is this test that is considered the WHO standard for malaria diagnosis. Regarding field diagnosis, the malaria case was defined by a positive result by microscopy and/or RDT.

### Microscopy examination

A thick drop smear was prepared from each capillary blood sample and stained with 10% Giemsa for 15 min [23]. The reading, diagnosis, and quantification of parasite load were performed as described by Febrer-Sendra et al. and by Alger et al. [23, 25]. Three groups of infection intensity were established according to the parasite load of each drop following the thresholds marked by Fox et al. [26], as follows: low intensity (< 800 parasites/µL), medium intensity (800–4000 parasites/µL), and high intensity (> 4000 parasites/µL). The detection limit of microscopic examination in this study was 80 parasites/µL.

### Rapid diagnostic test

The STANDARD™ Q Malaria Pf/Pan Ag test (SD Biosensor, Republic of Korea) was used for the rapid diagnosis of malaria (lot numbers: 5102A71AC/1, 5102BJ1AC1, and 5102C82AC1). This antigen test targets *Pf*HRP2 of *P. falciparum* and panmalaric lactate dehydrogenase (pLDH) of *P. falciparum*, *P. vivax*, *P. ovale,* and *P. malariae*. The detection limit is 163 parasites/µL. The test was carried out according to the manufacturer’s instructions.

### Molecular analysis

DNA extraction was performed on the same day of sample collection at the HNSP, using 200 µL of venous blood, and eluting DNA in 100 µL of elution buffer, as described by Febrer-Sendra et al. [23]. The eluates were stored in the HNSP laboratory at −20 °C until transport to Spain. Eluted DNA samples were shipped to the Microbiology Department of the Hospital Universitario Vall d'Hebron (HUVH, Barcelona, Spain) for analysis by real-time PCR. It should be noted that it was not possible to keep the samples cold during transport.

A first real-time PCR was performed to detect *Plasmodium* spp.-specific DNA using the RealStar^®^ Malaria PCR Kit 1.0 (Altona Diagnostics, Hamburg, Germany) according to the manufacturer’s instructions [limit of detection: 1.27; confidence interval (CI) 0.57–5.42 copies/µL of eluate]. Positive samples from the first PCR were tested in a second real-time PCR that detects *Plasmodium* species-specific DNA using the RealStar^®^ Malaria Screen & Type PCR Kit 1.0 (Altona Diagnostics), following the manufacturer’s instructions. This assay consists of two independent multiplex reactions, one targeting DNA specific to *P. falciparum* and *P. vivax* (limits of detection: 0.80 and 0.73; CI 0.44–2.45 and 0.46–1.62 copies/µL of eluate, respectively) and the other targeting DNA specific to *P. malariae*, *P. ovale*, and *P. knowlesi* (limits of detection: 0.36, 1.46, and 2.35; CI 0.24–0.74, 0.89–3.28, and 1.37–5.55 copies/µL of eluate, respectively). All real-time PCR reactions were performed using the CFX96 Touch real-time PCR detection system (Bio-Rad, Hercules, CA).

Negative (nuclease-free water) and positive (*Plasmodium* spp.-specific or species-specific DNA, obtained from DNA extraction from blood of patients with malaria attending HUVH, whose infection was confirmed by microscopy and real-time PCR) controls were included in each run. Samples were considered positive for *Plasmodium* DNA when the threshold cycle (Ct) for the *Plasmodium* target was < 40 and the internal control amplified effectively.

Although real-time PCR used in this study is not an absolute but a relative quantitative method, Ct values were used as an indirect estimate of parasite density [27].

### Statistical analysis

Qualitative variables were expressed as percentages and absolute frequencies. Quantitative variables were described using the mean or median and standard deviation (SD) or interquartile range (IQR) according to normality.

Spearman’s correlation test assessed the relationship between quantification via microscopy and real-time PCR. The point-biserial correlation test was utilized to establish the relationship between the patient’s age and diagnosis by real-time PCR. Potential associations between diagnosis using the different techniques, as well as participant’s gender in relation to real-time PCR diagnosis, were evaluated using the chi-squared test of independence or Fisher’s exact test. The Kruskal–Wallis test followed by the Mann–Whitney–Wilcoxon test, and single-factor analysis of variance (ANOVA) followed by Tukey’s test for multiple comparisons were applied to detect any significant differences between the groups established on the basis of infection intensity for quantification by microscopy and real-time PCR, respectively. To test whether there was an effect on parasite density due to coinfections, single-factor ANOVA and Tukey’s test for multiple comparisons were applied. In all cases, the significance level was set at *P*-value < 0.05.

Diagnostic performance parameters for each test (sensitivity, specificity, positive and negative predictive values) were calculated. Cohen’s Kappa coefficient was used to analyze the level of agreement between tests, and was interpreted as follows: slight agreement (0.00–0.20), fair agreement (0.21–0.40), moderate agreement (0.41–0.60), substantial agreement (0.61–0.80), and almost perfect agreement (0.81–1.00). Youden’s index was calculated to report the effectiveness of each technique: ineffective test (negative value) and effective test (positive value).

The excess risk of coinfections was calculated as described by Holzschuh et al. [21]. All species were assumed to be independently distributed; thus, if *X*_*i*_ is the frequency of one species and *X*_*j*_ of another, the expected frequency of a coinfection will be *X*_*i*_*X*_*j*_, and the excess risk will be $\frac{Xij}{XiXj}$, with *Xij* being the observed frequency of the coinfection [21].

Statistical analyses were performed using the R-UCA package for Windows (version R-UCA-3.3.1.exe).

## Results

### Characteristics of the study population

A total of 200 participants were included, of whom 54% (108/200) were female. In 199 cases, age could be recorded, with a median of 7 years (IQR 1.4–26.5), of which 43.7% (87/199) were PSAC, 19.6% (39/199) SAC, 29.7% (59/199) adults, and 7.0% (14/199) elderly.

### Malaria diagnosis and intensity of infection

Of all the febrile subjects, 58% (116/200) were diagnosed with malaria by at least one of the three techniques. Field diagnosis methods (microscopy and RDT) confirmed malaria in 51.5% (103/200) of cases. Regarding the technique used, 49% (98/200) of samples were positive by RDT, 39.5% (79/200) by real-time PCR, and 33.5% (67/200) by microscopy. Of the 98 RDT-positive cases, *Pf*HRP2 was detected in all of them, in 33.67% (33/98) of cases both *Pf*HRP2 and pLDH were detected, and no case was only positive for pLDH.

From the 67 microscopy-positive samples, the mean parasite density was 3120 parasites/µL (IQR 687–16,287): 47.8% (32/67) were classified as high, 22.4% (15/67) as moderate, and 29.9% (20/67) as low intensity.

The intensity of infection was estimated by real-time PCR, and the mean Ct value was 30.3 (SD 5.6). In cases positive by both microscopy and real-time PCR techniques, a correlation was observed between parasite density and Ct values (*r* = −0.5; *P*-value < 0.001).

When comparing microscopy and real-time PCR results, the high-intensity group continued to be the most represented (53.57%; 30/56). The median number of parasites in each group increased significantly (*P*-value < 0.05). As expected, lower Ct values were obtained in the high-intensity infection group. The median Ct of the high- and low-intensity groups presented statistically significant differences (*P*-value < 0.05) (Table 1).

**Table 1:** Intensity of *Plasmodium* spp. infection determined by microscopy and relationship with real-time PCR (*n* = 56)

Infection intensity	Infections	Microscopy	Real-time PCR
	*N* (%)	Parasite density (parasite/µL)^a^	Ct^b^
Low	12 (21.4)	314.0 (185.0–476.0) a^c^	32.9 (4.9) a^c^
Medium	14 (25.0)	1920.0 (1255.3–2523.8) b^c^	29.5 (4.5) ab^c^
High	30 (53.6)	18,121.5 (9779.3–81,959.5) c^c^	26.4 (5.28) b^c^

^a^The results are expressed as the median and its interquartile range

^b^The results are expressed as the mean and its standard deviation

^c^Letters indicate statistically significant differences between groups of different infection intensities for each of the diagnostic techniques (*P*-value < 0.05)

### RDT, real-time PCR, and microscopy results by age and gender

The median age of participants diagnosed with malaria by at least one of the techniques employed was 4 years (IQR 1.3–15.5). The technique with the lowest median age for positive cases was RDT (median = 3; IQR 1.2–10.0). The results of the three techniques employed were significantly related to age (*P*-value < 0.05) (Table 2). The group with the highest proportion of positive results by real-time PCR was SAC (48.7%, 19/39), however, no statistically significant association was found between the different age groups established (*P*-value > 0.05) (Table 2).

**Table 2:** Demographic data of the participant’s positive for the different diagnostic methods used

Demographic data		RDT		Real-time PCR		Microscopy		RDT ± real-time PCR ± microscopy^a^	
		*N* (%)	*P*-value (*r*)^c^	*N* (%)	*P*-value (*r*)^c^	*N* (%)	*P*-value (*r*)^c^	*N* (%)	*P*-value (*r*)^c^
Age	Age (years)^b^	3 (1.2–10.0)	< 0.001* (−0.37)	5 (1.7–17)	< 0.05* (−0.15)	5 (1.5–14.8)	< 0.05* (−0.16)	4 (1.3–15.5)	< 0.001* (−0.27)
	0–4.9 years (87/199; 43.7%)	54 (62.1)	< 0.001*	37 (42.5)	> 0.05	32 (36.8)	> 0.05	58 (66.7)	< 0.01*
	5–14.9 years (39/199; 19.6%)	25 (64.1)		19 (48.7)		17 (43.6)		27 (69.2)	
	15–49.9 years (59/199; 29.7%)	15 (25.4)		18 (30.5)		14 (23.7)		25 (42.4)	
	> 50 years (14/199; 7.0%)	3 (21.4)		5 (35.7)		3 (21.4)		5 (35.7)	
Gender	Female (108/200; 54%)	55 (50.9)	> 0.05	47 (43.5)	> 0.05	41 (38.0)	> 0.05	66 (61.1)	> 0.05
	Male (92/200; 46%)	43 (46.7)		32 (34.8)		26 (28.3)		50 (54.3)	

^a^Patients with a positive result in at least one of the three techniques (RDT, real-time PCR, and/or microscopy)

^b^The results are expressed as the median and its interquartile range

^c^The *r*-value is the Pearson correlation coefficient of age with respect to malaria diagnosis by the different techniques.

*Statistically significant

Women presented a higher proportion of positive cases detected by all techniques, however, no significant association was found with the gender of the participants (*P*-value > 0.05) (Table 2).

## Performance of diagnostic methods

When microscopy is considered the reference technique, the sensitivity of real-time PCR was 83.6% with a specificity of 82.7%. Both techniques showed a substantial level of agreement (*κ* = 0.64) (Table 3). When using the field diagnosis as the reference (microscopy and/or RDT), real-time PCR had a sensitivity of 64.1% with a specificity of 86.6%, showing moderate agreement (*κ* = 0.5) between both methods (Table 4). In contrast, using a positive result of at least one of malaria diagnosis techniques (RDT, real-time PCR, and/or microscopy) as the reference, the sensitivity of RDT was 84.5%, that of real-time PCR 68.1%, and that of microscopy 57.8% (Table 5).

**Table 3:** Performance of real-time PCR using microscopy as the reference (*n* = 200)

		Microscopy *N*		Sensibility (95% CI)^a^	Specificity (95% CI)^a^	PPV^a^ (95% CI)^a^	NPV^a^ (95% CI)^a^	Kappa (95% CI)^a^	Youden’s index
		+	−						
Real-time PCR	+	56	23	83.6 (72.1–91.1)	82.7 (75.0–88.5)	70.9 (59.4–80.3)	90.9 (84.0–95.2)	0.64 (0.52–0.75)	0.66
	−	11	110						

^a^CI, confidence interval; PPV, positive predictive value; NPV, negative predictive value

**Table 4:** Performance of real-time PCR using field diagnosis (microscopy and RDT) as the reference (*n* = 200)

		Microscopy ± RDT^a^ *N*		Sensibility (95% CI)^b^	Specificity (95% CI)^b^	PPV^b^ (95% CI)^b^	NPV^b^ (95% CI)^b^	*κ* (95% CI)^b^	Youden’s index
		+	−						
Real-time PCR	+	66	13	64.1 (54.0–73.1)	86.6 (77.8–92.4)	83.5 (73.1–90.6)	69.4 (60.3–77.3)	0.50 (0.38–0.62)	0.50
	−	37	84						

^a^Patients with a positive result in at least one of the two techniques used in the field (microscopy and/or RDT)

^b^CI, confidence interval; PPV, positive predictive value; NPV, negative predictive value

**Table 5:** Performance and comparative results of malaria diagnostic techniques using all malaria diagnosis techniques as the reference (*n* = 200)

		RDT ± real-time PCR ± microscopy^a^ *N*		Sensibility (95% CI)^b^	Specificity (95% CI)^b^	PPV^b^ (95% CI)^b^	NPV^b^ (95% CI)^b^	Youden’s Index
		+	−					
RDT	+	98	0	84.5 (76.8–90)	100 (95.6–100)	100 (96.2–100)	84.2 (73.8–88.5)	0.85
	−	18	84					
Real-time PCR	+	79	0	68.1 (59.2–75.9)	100 (95.6–100)	100 (95.4–100)	69.4 (60.7–76.9)	0.68
	−	37	84					
Microscopy	+	67	0	57.8 (48.7–66.4)	100 (95.6–100)	100 (94.6–100)	63.2 (54.7–70.9)	0.58
	−	48	84					

^a^Patients with a positive result in at least one of the three techniques (RDT, real-time PCR, and/or microscopy)

^b^CI, confidence interval; PPV, positive predictive value; NPV, negative predictive value

**Fig. 2:**
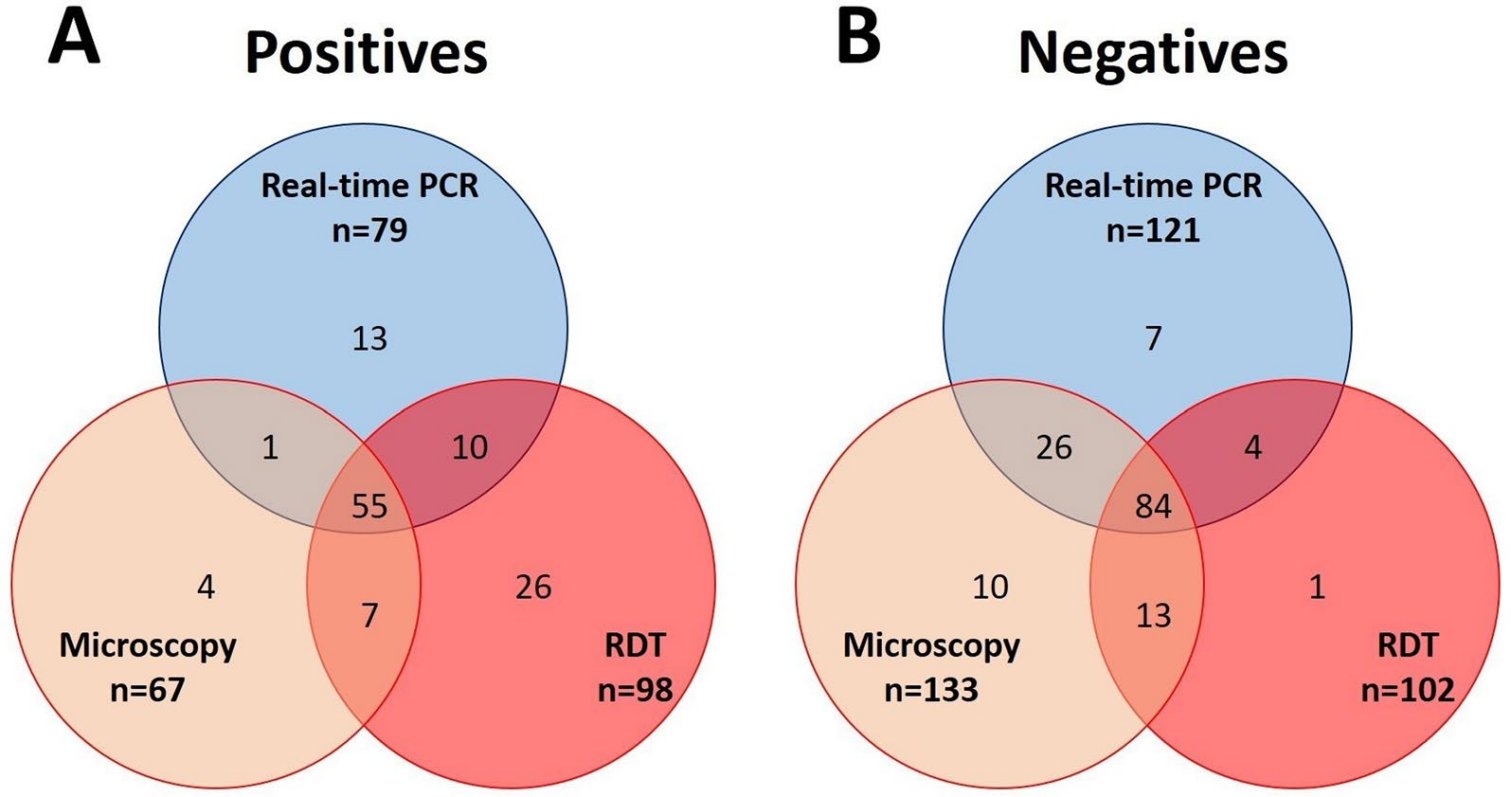
Venn diagrams for comparison between diagnostic techniques (real-time PCR, microscopy, and RDT). **A** Distribution of positive samples for *Plasmodium* spp. infection for each technique. **B** Distribution of negative samples for *Plasmodium* spp. infection for each technique

The similarities and discrepancies between the diagnostic techniques are shown in Fig. 2. A total of 55 samples were positive and 84 negative by the three diagnostic techniques (real-time PCR, RDT, and microscopy). The highest similarity of positive cases was observed between real-time PCR and RDT (10 samples), while the negative cases for infection showed higher similarities in the diagnosis by real-time PCR and microscopy (26 samples). Despite the discordances, malaria diagnosis by each of the techniques was shown to be significantly dependent (*P*-value < 0.001).

### Molecular identification of *Plasmodium* species

**Fig. 3:**
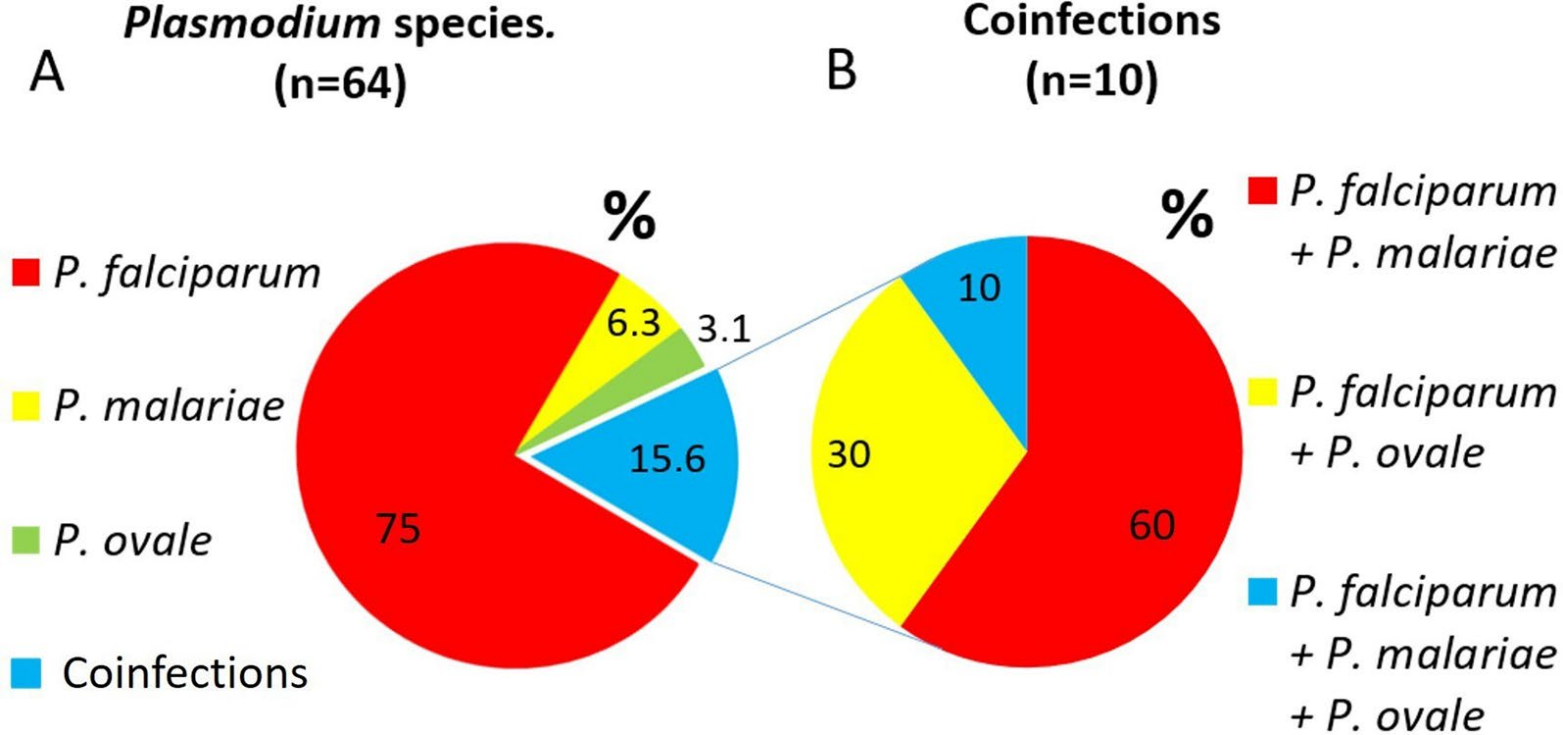
Molecular identification of *Plasmodium* species in patients diagnosed with malaria by real-time PCR. **A** The results of real-time PCR specific to each *Plasmodium* species are shown graphically. Coinfections refer to infection by more than one *Plasmodium* species. Results are expressed as the percentage of infections in which the species could be determined (*n* = 64). **B** Coinfections of different *Plasmodium* species are plotted. Results are expressed as a percentage of coinfections (*n* = 10)

Species identification was not successful in 19% (15/79) of the confirmed cases due to low sample parasitaemia (first real-time PCR Ct values > 34.5). The species could be identified in 64 samples, with *P. falciparum* being the most prevalent (90.6%; 58/64), followed by *P. malariae* (17.2%; 11/64) and *P. ovale* (9.4%; 6/64). Single infection was determined in 84.4% (54/64) of cases, while coinfections with two or more *Plasmodium* species were found in 15.6% (10/64) (Fig. 3a).

Of all monoinfections, those caused by *P. falciparum* were by far the most prevalent (88.9%; 48/54), followed by *P. malariae* (7.4%; 4/54) and *P. ovale* (3.7%; 2/54). In these cases, 83.3% (45/54) were RDT-positive (42 *P. falciparum*, 2 *P. malariae*, and 1 *P. ovale*). *Pf*HRP2 was detected in all positive RDTs; in contrast, the pLDH band was only visualized in 57.14% (24/42) of *P. falciparum* infections and one case of *P. ovale* but not in *P. malariae*. Of patients with *P. falciparum* monoinfections, 45.8% (22/48) were PSAC, 25% (12/48) SAC, 22.9% (11/48), and 6.3% (3/48) elderly; of those monoinfected with *P. malariae*, 50% (2/4) were PSAC, 25% (1/4) SAC, and 25% (1/4) elderly, while those monoinfected with *P. ovale* were all SAC.

Regarding the ten coinfections, *P. falciparum* was present in all cases, with *P. falciparum* + *P. malariae* being the most common combination (60%; 6/10), followed by *P. falciparum* + *P. ovale* (30%; 3/10). One case of triple coinfection with *P. falciparum* + *P. malariae* + *P. ovale* was found (10%; 1/10) (Fig. 3b). All cases of coinfection were RDT-positive. *Pf*HRP2 bands were visualized in all these cases, whereas pLDH was visualized in 66.7% (4/6) of *P. falciparum* + *P. malariae* coinfections and 33.3% (1/3) of *P. falciparum* + *P. ovale*, but not in the case of triple coinfection. Patients with coinfections were 6/10 (60%) PSAC, 3/10 (30%) SAC, and only 1/10 (10%) was an adult, aged 17 years.

**Fig. 4:**
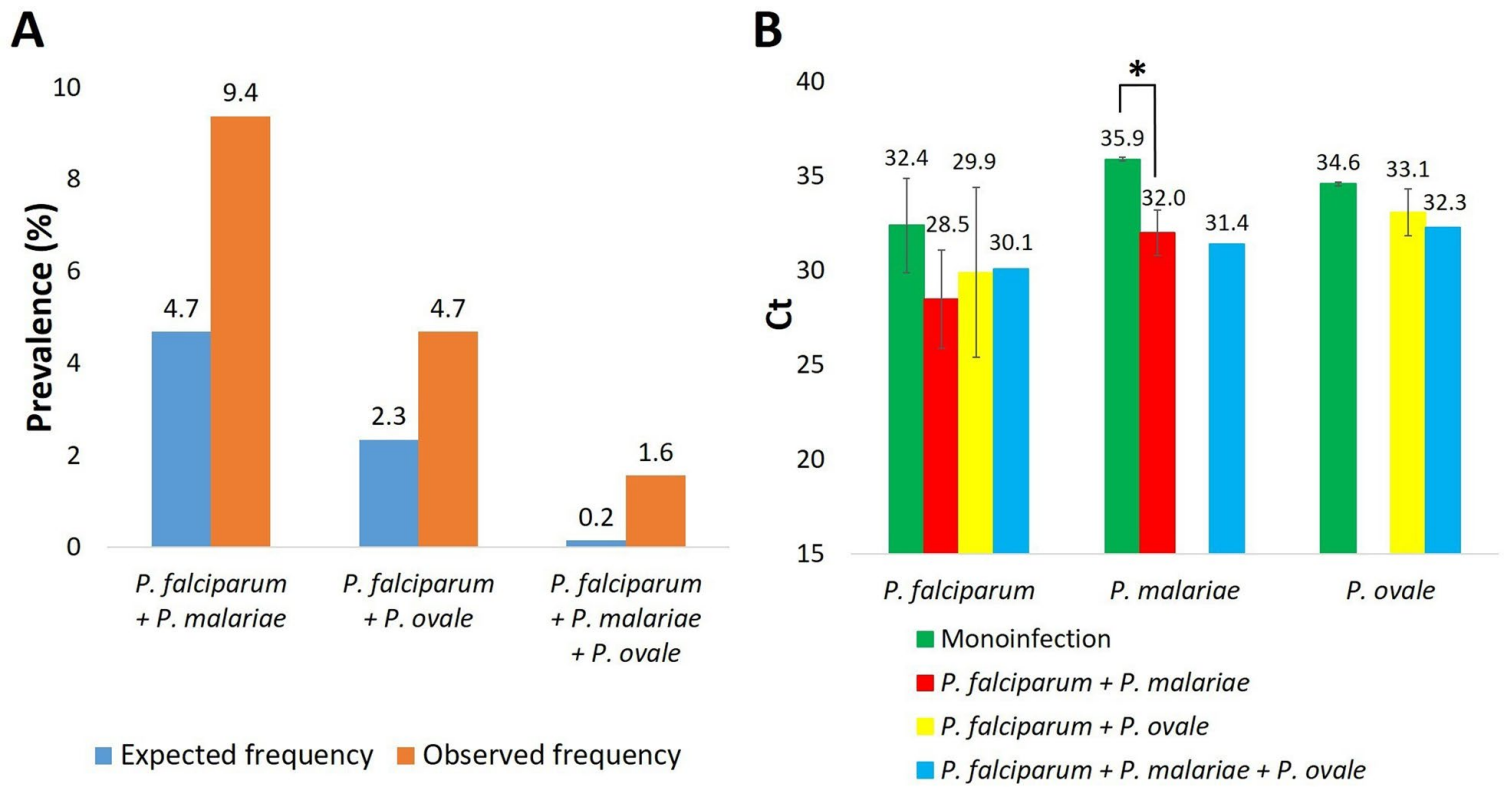
Prevalence of *Plasmodium* coinfections and effect of coinfection on parasite load estimated by real-time PCR. **A** Expected and actual prevalence of different coinfections with the three *Plasmodium* species detected by real-time PCR. **B** Relative quantification by real-time PCR of the density of each *Plasmodium* species according to the type of infection (mono or coinfection). Results are expressed as mean Ct and SD (error bars). The asterisk indicates significant differences (*P*-value < 0.05) between the mean Ct of *P. malariae* infections in the case of monoinfections and coinfection with *P. falciparum*

The expected and observed frequencies of the different coinfections were as follows: 4.7% and 9.4% of *P. falciparum* + *P. malariae*, 2.3% and 4.7% of *P. falciparum* + *P. ovale*, and 0.2% and 1.6% of triple coinfection, respectively (Fig. 4a). The excess risk was estimated for the coinfections: triple infection (10.7), *P. falciparum* + *P. malariae* (2.0), and *P. falciparum* + *P. ovale* (2.0).

The results of the possible effect of coinfections on parasite density estimated by real-time PCR are shown in Fig. 4b. The mean Ct value for *P. falciparum* was lower when coinfected with *P. malariae* (Ct = 28.5) compared with single infection (Ct = 32.4) (*P*-value > 0.05). The same was observed for *P. malariae* coinfected with *P. falciparum* (Ct = 32.0) compared with monoinfection (Ct = 35.9) (*P*-value < 0.05).

## Discussion

The aim of this study was to evaluate the usefulness of real-time PCR for the diagnosis of malaria in a group of febrile patients from a rural setting in Angola. The prevalence of malaria in patients with febrile syndrome in Cubal was 58%. In a study conducted in Bengo (a province in northwestern Angola), the prevalence observed was 15.9%, determined by microscopy and PCR [28]. These differences were attributed to the fact that in the Bengo study, participants were selected regardless of their symptomatology. Other studies conducted in localities of Central African countries such as Gabon and the Republic of the Congo, where the transmission rate is higher and more uniform than in Angola, showed a malaria prevalence of 69.6% in febrile children by PCR and 32.5% in febrile patients of all ages by RDT, respectively [29, 30].

RDTs and microscopy are the most widely used diagnostic methods for malaria control [31]. Microscopy remains the gold standard for the diagnosis of malaria infection; however, more sensitive methods capable of providing early and accurate diagnosis are needed [32]. In this study, taking the results of three techniques used as the reference, real-time PCR had a higher sensitivity (68.1%) than microscopy (57.8%) but RDT had a higher sensitivity (84.5%) and negative predictive value than real-time PCR. However, using microscopy as the reference method, real-time PCR showed a higher specificity and positive predictive value than RDT according to the results published by Febrer-Sendra et al. for RDT [23].

Our results agree with other published works in which real-time PCR and RDT had higher sensitivities than microscopy, taking the results of the three techniques as the reference [33–36]. In contrast, most of them obtained a higher sensitivity for real-time PCR than RDT [34, 36]. However, other studies obtained similar sensitivities [35], and others agreed with our results [33]. In our case, this low sensitivity of real-time PCR could be due to inadequate storage and transport of DNA eluates, which could have caused sample degradation. In addition, the use of venous blood for DNA extraction may have reduced the sensitivity of real-time PCR instead of using capillary blood [37]. 

Classic microscopy is considered the gold standard method for malaria diagnosis, and RDT and real-time PCR are more sensitive alternatives for estimating the prevalence in patients with febrile symptoms in endemic areas [35]; however, PCR has not yet been adequately implemented in the field. In this sense, LAMP is a good alternative in rural areas with limited resources. In the study by Febrer-Sendra et al. conducted in Cubal in 2022, a colorimetric and real-time LAMP was used for malaria diagnosis, obtaining a higher performance than that obtained in this study using real-time PCR [23]. These results suggest the importance of implementing a diagnostic method with better performance than microscopy. Therefore, and despite the effort made in this study, the strategy of performing DNA extraction *in situ* is not recommended unless optimal storage conditions can be guaranteed for a truly effective molecular analysis.

Regarding the discordances obtained between the three diagnostic methods, samples that were positive by microscopy or RDT and negative by real-time PCR could be explained by the different method of blood collection and the possible degradation of the DNA during transport to Barcelona. On the contrary, those that were positive by real-time PCR and negative by microscopy or RDT could be due to the parasite load being submicroscopic or below the RDT detection limit. Nonetheless, there is a possibility that these latter mismatches between real-time PCR and RDT could be due to a deletion of the *pfhrp2* gene (which encodes for the target protein of the antigenic test), as has been reported in other countries in West sub-Saharan Africa [38, 39].

Furthermore, quantification of parasite density by microscopy provides information about the intensity of the infection as well as monitoring the response to treatment [40]. In addition, real-time PCR allows for an indirect estimation of the parasite load through Ct values [41]. It was expected that most febrile patients show a high intensity of infection, as published by Mangal et al. [42]. In our case, we observed that as parasite densities determined by microscopy increased, the Ct of real-time PCR decreased. Thus, this Ct value could be an indirect measure of parasite load and would allow the determination of the intensity of infection, at least in the high- and low-intensity groups.

Gender was not related to real-time PCR diagnosis, however, female patients and children presented a higher proportion of positive cases, as described in another study carried out in Ethiopia [43]. These results highlight the great importance of focusing on the younger age groups, as children under 5 years of age are those most affected by malaria worldwide and have the highest mortality. In our case, the technique with the greatest capacity to identify infections at an earlier age was RDT, with a higher correlation than real-time PCR or microscopy. Again, RDT yielded better results than real-time PCR. Moreover, its rapidity in producing a result makes it a highly effective technique for the diagnosis of malaria in children. Nevertheless, once again we emphasize the importance of sample preservation during transport, which could have altered the results of real-time PCR.

Single-step real-time multiplex PCR has advantages over end-time PCR, such as greater sensitivity, speed, and lower risk of cross-contamination, in addition to allowing for the identification of *Plasmodium* species in fewer reactions [44]. In our study, the most prevalent *Plasmodium* species was *P. falciparum*, followed by *P. malariae* and *P. ovale*. The same results were previously described by Fançony et al. in Northern Angola in 2012 [3].

It is important to highlight that coinfections with different *Plasmodium* species are frequently overlooked by microscopic examination [12]. In this study, a higher number of mixed infections (15.6%) was found compared with those reported by other studies conducted in Angola (7.6%) or Zambia (10.3%) [3, 45]. Regarding causative species, the most prevalent coinfection was caused by *P. falciparum* and *P. malariae,* in concordance with published data from Angola and Zambia [3, 45].

Since *P. falciparum* is the most prevalent species in Cubal, and it was expected to be present in all cases of coinfection. On the basis of our results, we found that the prevalence of coinfections was higher than expected if each species was randomly distributed in the host population. Therefore, we believe that there must be one or more factors that could explain this fact, such as different host susceptibility to infection and/or heterogeneity of individual exposure to mosquito bites [21]. It should be noted that most coinfected patients were children, and only one corresponded to an adult (17 years old).

From the real-time PCR Ct values, we were able to estimate the effect of coinfections on the parasite density of each *Plasmodium* species. Interestingly, when *P. malariae* coinfected with *P. falciparum*, the Ct values of *P. malariae* were lower than in individual infection, which implies that its parasite density is higher when coinfected with *P. falciparum*. Our results are in perfect agreement with those of another study in Papua New Guinea, in which they observed this benefit of *P. malariae* when coinfected with other species [21]. An increase in the parasite density of *P. falciparum* and *P. ovale* was also observed in coinfections; however, the low number of samples could explain why a statistical relationship in these cases was not observed.

A striking fact of this study is that three patients infected only with non-*P. falciparum* species (two *P. malariae* and one *P. ovale*) were positive for the detection of *Pf*HRP2 by RDT. As this protein is specific to *P. falciparum*, these results would support the idea that the DNA could be degraded during sample transport, with the consequent non-detection of *P. falciparum*.

### Limitations of this study

Several limitations were identified in this study that could affect the interpretation of the results. Microscopy was performed by a single microscopist rather than by two independent technicians, which limits the robustness of the results. It is also important to note that the blood sample used for microscopy and RDT diagnosis (capillary blood) was not the same as that used for real-time PCR (whole blood), which could introduce variability in the results. During the transport of the eluted DNA to the HUVH in Barcelona, the cold chain could not be maintained at all times, which could have affected the quality of the DNA analyzed. Finally, the positive controls used in the PCRs did not correspond to strains commonly used in this type of study, such as 3D7 or NF54, but were based on DNA from the blood of patients previously diagnosed with malaria by microscopy and real-time PCR, which could limit the comparability of the results.

## Conclusions

Despite logistical constraints regarding the preservation of DNA eluates during transport, our study demonstrated that real-time PCR is more effective than microscopy for detecting malaria in febrile patients in Cubal (Angola). It is imperative that the method of sampling and preservation during transport be rigorously adhered to so that an accurate result of molecular techniques can be ensured. Furthermore, real-time PCR can provide an estimate of the intensity of infection and allows for the identification of *Plasmodium* species for surveillance purposes.

## Data Availability

The data supporting the conclusions of this article are included in this manuscript.

## Abbreviations

CI: Confidence interval
Ct: Threshold cycle
HNSP: Hospital Nossa Senhora da Paz
HUVH: Hospital Universitario Vall d'Hebron
IQR: Interquartile range
LAMP: Loop-mediated isothermal amplification
PCR: Polymerase chain reaction
*Pf*HRP2: Histidine-rich protein 2 of *P. falciparum*
pLDH: Panmalaric lactate dehydrogenase
PSAC: Preschool-aged children
RDT: Rapid diagnostic test
SAC: School-aged children
SD: Standard deviation
WHO: World Health Organization
